# LDC7559 inhibits microglial activation and GSDMD-dependent pyroptosis after subarachnoid hemorrhage

**DOI:** 10.3389/fimmu.2023.1117310

**Published:** 2023-03-29

**Authors:** Wenhua Cai, Zhe Wu, Jinqing Lai, Jieran Yao, Yile Zeng, Zhongning Fang, Weibin Lin, Junyan Chen, Chaoyang Xu, Xiangrong Chen

**Affiliations:** ^1^ Department of Neurosurgery, The Jinjiang Municipal Hospital, Quanzhou, Fujian, China; ^2^ Department of Neurosurgery, The Second Affiliated Hospital of Fujian Medical University, Quanzhou, Fujian, China; ^3^ Centre of Neurological and Metabolic Research, The Second Affiliated Hospital of Fujian Medical University, Quanzhou, Fujian, China

**Keywords:** subarachnoid hemorrhage, microglia, pyroptosis, LDC7559, GSDMD

## Abstract

Mounting evidence indicates that inhibition of microglial activation and neuronal pyroptosis plays important roles in brain function recovery after subarachnoid hemorrhage (SAH). LDC7559 is a newly discovered gasdermin D (GSDMD) inhibitor. Previous studies have demonstrated that LDC7559 could inhibit microglial proliferation and pyroptosis. However, the beneficial effects of LDC7559 on SAH remain obscure. Based on this background, we investigated the potential role and the mechanism of LDC7559 on SAH-induced brain damage both *in vivo* and *in vitro*. The findings revealed that microglial activation and neuronal pyroptosis were evidently increased after SAH, which could be markedly suppressed by LDC7559 both *in vivo* and *in vitro.* Meanwhile, LDC7559 treatment reduced neuronal apoptosis and improved behavior function. Mechanistically, LDC7559 decreased the levels of GSDMD and cleaved GSDMD after SAH. In contrast, nod-like receptor pyrin domain-containing 3 (NLRP3) inflammasome activation by nigericin increased GSDMD-mediated pyroptosis and abated the beneficial effects of LDC7559 on SAH-induced brain damage. However, LDC7559 treatment did not significantly affect the expression of NLRP3 after SAH. Taken together, LDC7559 might suppress neuronal pyroptosis and microglial activation after SAH by inhibiting GSDMD, thereby promoting brain functional recovery.

## 1 Introduction

Subarachnoid hemorrhage (SAH) is still a catastrophic stroke with high morbidity rate (1). Although therapeutic interventions for SAH have made great progress, most SAH survivors still experience long-term physical and neurological deficits. SAH involves a variety of pathogenic mechanisms. A considerable number of preclinical and clinical studies have observed that the early robust neuroinflammation might explain the poor outcome after SAH (2–6). Microglia, the primary innate immune cells in the brain, plays a critical role in immune response. After hemorrhage, microglial activation can induce the release of pro-inflammatory mediators to further exacerbate neuroinflammation (7, 8). Thus, diminishing cerebral inflammation and inhibiting microglial activation would improve brain recovery after SAH, but effective therapies are lacking.

Evidence indicates that different forms of cell death can occur in pathological processes of inflammatory response. In addition to common cell death, pyroptosis can be driven by inflammatory processes (9, 10). Gasdermin D (GSDMD) is the inducer of pyroptosis. Activated inflammatory caspases can cleave GSDMD and release an amino (N)-terminal effector domain (GSDMD-N). GSDMD-N could rupture cell membranes and induce membrane pore formation and inflammatory cytokines secretion (11). Nod-like receptor pyrin domain-containing 3 (NLRP3) inflammasome is the currently best studied inflammasome in various disease models. Upon stimulation, NLRP3 inflammasome activation could trigger caspase-1 to cleave GSDMD, thereby initiating GSDMD-N-mediated pyroptosis (12–14). In SAH area, mounting evidence has indicated that inhibiting NLRP3 inflammasome and pyroptosis could improve neurological outcomes after SAH (15–18). These suggest that inhibiting NLRP3/caspase-1/GSDMD might be a means to treat SAH.

LDC7559 is a newly GSDMD selective inhibitor. Previous studies have indicated that LDC7559 could block the GSDMD-N domain, thereby suppressing inflammation (19, 20). A recent study by Yu et al. reported that LDC7559 can suppress pyroptosis and inhibit neuroinflammation by suppressing GSDMD after traumatic brain injury (TBI) both *in vivo* and *in vitro (* 21). However, the potential role of LDC7559 in SAH remains unknown. Based on this background, we tried to explore whether LDC7559 can inhibit microglial activation and pyroptosis after SAH and the potential mechanisms both *in vivo* and *in vitro* SAH models.

## 2 Materials and methods

### 2.1 Animals and *in vivo* model

Adult male Sprague-Dawley rats (weighing 230-280 g) were used in our study. Rat model of SAH was built in accordance with previous published method (22). Before SAH, anesthetization was administered with 1% sodium pentobarbital. A marked filament (4-0) was used to puncture the origin of the left middle cerebral artery. Sham-operated rats received the same treatment, but not with vessel perforation. *In vivo* experiments, rats were randomly allotted to sham group (n= 18), SAH + vehicle group (n= 22, 4 animals died), SAH + LDC7559 group (n= 60, 6 animals died), and SAH + LDC7559 + nigericin group (n=21, 3 animals died). Investigators blinded to the experimental groups performed experimental analyses.

### 2.2 Cell culture

Primary neurons and microglia were dissociated from rat pups (postnatal days 0-1) and cultured as previously described (23). Primary cortical neurons were seeded in poly-D-lysine-coated plates and maintained in neurobasal media containing B27 and glutamate. Primary microglia were cultured in Dulbecco’s modified Eagle Medium (DMEM, GIBCO). The co-culture system was supplemented with DMEM with 10% FBS. *In vitro* experiments, primary cortical neurons and microglia were exposed to 10 μM hemoglobin (Hb).

### 2.3 Drug administration

*In vivo*, LDC7559 (MedChemExpress) was diluted in 10% DMSO and 90% corn oil before use. LDC7559 (10, 20, or 30 mg/kg) or vehicle was treated at 2 h after SAH by intraperitoneally administration and then once a day. Nigericin (MedChemExpress, 2 μg) was prepared in 2 μl ethanol and physiologic saline and was intracerebroventricularly administered at 2 h before SAH. For experiments *in vitro*, three different doses of LDC7559 (5, 25, or 50 μM) was dissolved in culture medium. Nigericin (5 µM) was added into culture medium after Hb stimulation. The concentrations and administration routes of LDC7559 and nigericin were based on previous studies (21, 24).

### 2.4 Cell viability assay

A commercial cell counting kit-8 (CCK-8) assay was used to detect cell viability. According to the instructions (Beyotime Biotechnology, China), cells were incubated with CCK-8 reagent. The optical density was measured at 450 nm.

### 2.5 Tissue processing

After rats were deeply anesthetized, the animals were transcardially perfused with phosphate buffer solution at room temperature. The fresh inferior basal temporal lobe was employed for western blotting and ELISA assay. For histological studies, brains were immersed in 4% paraformaldehyde overnight. After brain samples were transferred sequentially into different sucrose solution (15%, 25% and 35%), they were sectioned into 8-μm coronal sections. The brain sections were kept in refrigerater at -20°C.

### 2.6 ELISA assay

After sample preparation, we determined the expressions of IL-1β, IL-6, and IL-18 using the respective commercial kits (Multi Sciences. China). The expressions of these cytokines were calculated based on the instructions.

### 2.7 Western blot analysis

After sample preparation, the same mass of sample was separated by SDS-PAGE gels. After they were transferred to PVDF membrane, the membranes were incubated with diluted specific antibodies overnight at 4°C. Then, they were incubated with appropriate secondary antibodies. Protein signals were analyzed with Image J software. Antibodies used for western blot can be seen in **Supplementary Material**.

### 2.8 Immunofluorescence staining

Immunofluorescence staining was carried out by standard procedures (25, 26). Briefly, frozen brain sections and cells were fixed with 4% paraformaldehyde. After blocking, the slides were stained with primary antibodies and appropriate secondary antibodies. 4,6-diamidino- 2-phenylindole (DAPI) was employed to indicate the nuclei. A fluorescence microscope was employed for fluorescence detection. Antibodies used for immunofluorescence staining can be seen in **Supplementary Material**.

### 2.9 TUNEL assay

TUNEL was conducted in line with the manufacturer’s protocol (Beyotime Biotechnology). Frozen brain sections and cells were incubated with the NeuN antibody, and then with a TUNEL reaction solution. After washing, they were stained with DAPI. Brain sections and cells were observed with a fluorescence microscope.

### 2.10 Behavior function

The neurologic deficits of SAH rats were determined by using a previous published method (27). A lower score suggests more severe deficits. To further evaluate the motor deficits after SAH, the beam-balance score test was performed according to previous studies (6). Behavior functions were recorded in a blinded manner.

### 2.11 Statistics

All data are expressed as mean ± SD. Comparisons between groups were determined by one-way analysis of variance with Bonferroni *post hoc* test. The GraphPad Prism 8 software was employed for all statistical analysis. Probability value <0.05 was considered significantly different.

## 3 Results

### 3.1 LDC7559 treatment mitigated neurological impairment after SAH

**Figure 1:**
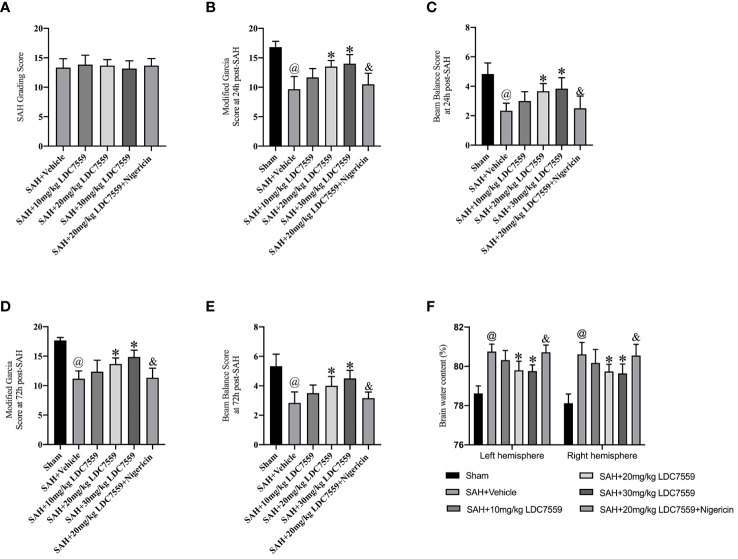
LDC7559 improved short-term neurological outcome after SAH. **(A)** SAH grading scores in all experimental groups (n = 6 per group). Quantification of the modified Garcia score **(B)** and beam-walking test **(C)** at 24 h post-SAH (n = 6 per group). Quantification of the modified Garcia score **(D)** and beam-walking test **(E)** at 72 h post-SAH (n = 6 per group). Measurement of brain edema in all groups at 24 h post-SAH **(F)** (n = 6 per group). Values are presented as the mean ± SD. ^@^ *P* < 0.05 compared with sham group, *P < 0.05 compared with SAH + vehicle group, ^&^ *P* < 0.05 compared with SAH + 20mg/kg LDC7559 group.

There was no significant difference in the average SAH grading scores (**Figure 1A**). The Modified Garcia test and beam-walking test were used to determine the effects of LDC7559 on short-term neurological impairments. As shown, SAH insults induced a significant neurological deterioration compared with the sham group. LDC7559 treatment dose-dependently improved neurological deficits and motor function compared with the vehicle-treated SAH group (**Figures 1B–E**). In addition, brain water content showed that LDC7559 treatment at both 20 mg/kg and 30 mg/kg significantly reduced brain edema as compared with the SAH group (**Figure 1F**). Based on these results, 20 mg/kg LDC7559 was adopted as the optimum dose. Nigericin, a potent NLRP3 activator, was further employed in this experiment. Our data further revealed that nigericin aggravated neurological impairments and brain edema after SAH and abated the cerebroprotective effects of LDC7559 against SAH (**Figures 1B–E**).

### 3.2 LDC7559 treatment inhibited microglial activation and inflammatory response following SAH

**Figure 2:**
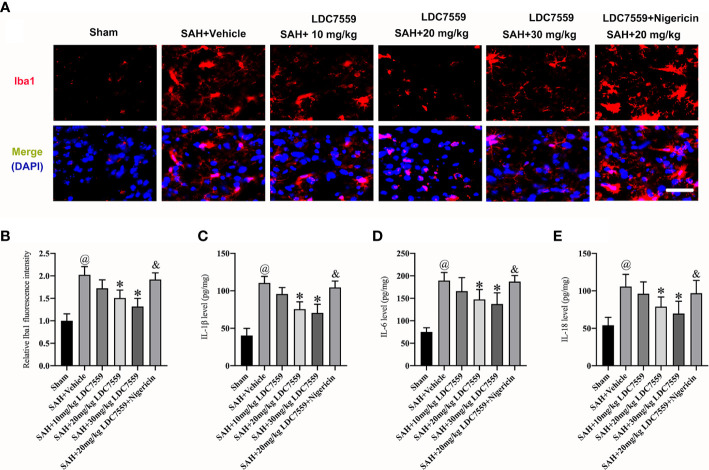
LDC7559 mitigated microglial activation and neuroinflammation after SAH. Representative brain sections **(A)** and measurement of Iba-1 staining **(B)** (n = 6 per group). Scale bar = 50 μm. Measurement of IL-1β **(C)**, IL-6 **(D)**, and IL-18 **(E)** expressions (n = 6 per group). Values are presented as the mean ± SD. ^@^ *P* < 0.05 compared with sham group, *P < 0.05 compared with SAH + vehicle group, ^&^ *P* < 0.05 compared with SAH + 20mg/kg LDC7559 group.

Microglial activation is essential to neuroinflammation after SAH. A previous study reported that LDC7559 can inhibit microglial proliferation and activation after TBI (21). In time, the pro-inflammatory cytokines release after TBI were decreased by LDC7559. We speculated that LDC7559 can also ameliorate microglial activation as well as the subsequent inflammatory response. The immunofluorescence staining results showed that microglial activation was significantly induced after SAH, which could be dose-dependently suppressed by LDC7559 (**Figures 2A, B**). In addition, SAH insults markedly triggered the levels of IL-1β, IL-6, and IL-18 in the brain samples, which could be decreased by LDC7559 (**Figures 2C–E**). In contrast, nigericin further aggravated microglial activation and abated the anti-inflammatory effects of LDC7559 against SAH (**Figures 2A–E**).

### 3.3 LDC7559 treatment inhibited neuronal pyroptosis and neuronal apoptosis following SAH

**Figure 3:**
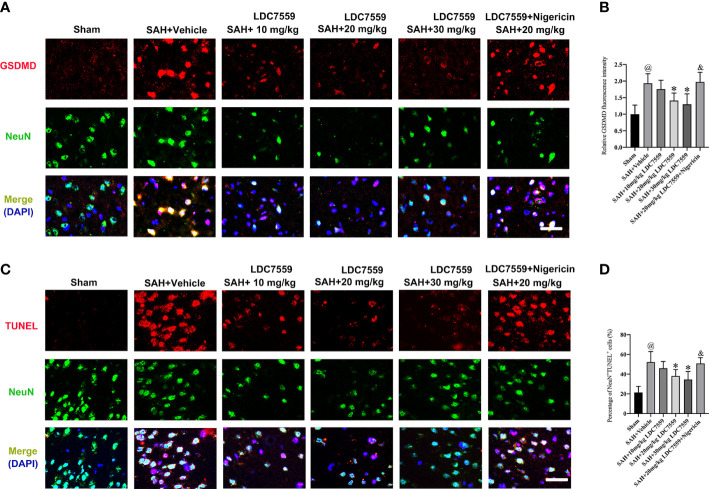
LDC7559 mitigated neuronal apoptosis and pyroptosis following SAH. Representative immunofluorescence microphotographs of GSDMD-positive neurons **(A)** in the ipsilateral hemisphere. Scale bar = 50 μm. Measurement of GSDMD-positive neurons in the ipsilateral hemisphere **(B)** (n = 6 per group). Representative immunofluorescence microphotographs of TUNEL-positive neurons **(C)** in the ipsilateral hemisphere. Scale bar = 50 μm. Measurement of TUNEL-positive neurons in the ipsilateral hemisphere **(D)** (n = 6 per group). Values are presented as the mean ± SD. ^@^ *P* < 0.05 compared with sham group, *P < 0.05 compared with SAH + vehicle group, ^&^ *P* < 0.05 compared with SAH + 20mg/kg LDC7559 group.

Evidence indicates that neuronal apoptosis and neuronal pyroptosis play determinant roles in the poor outcome after SAH. Pyroptosis is a recently discovered cell death form, which can be driven by inflammatory processes. Studies have demonstrated that pyroptosis is characterized by GSDMD-mediated. Inhibiting neuronal apoptosis and neuronal pyroptosis have been shown to improve neurological outcomes after SAH. LDC7559 is a newly selective GSDMD inhibitor. We then evaluated the influence of LDC7559 on neuronal apoptosis and pyroptosis. The data revealed that SAH significantly induced the number of GSDMD-positive and TUNEL-positive neurons, which could be markedly inhibited after LDC7559 (**Figures 3A–D**). However, the beneficial actions of LDC7559 on neuronal apoptosis and neuronal pyroptosis were abated by nigericin administration (**Figures 3A–D**).

### 3.4 LDC7559 treatment inhibited GSDMD-mediated signaling after SAH

**Figure 4:**
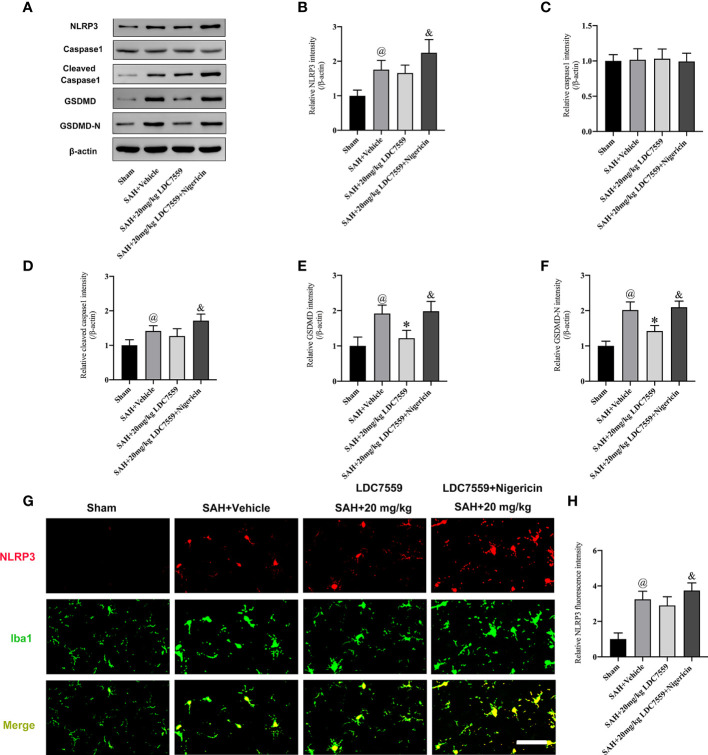
LDC7559 Treatment Inhibited GSDMD-mediated Signaling after SAH. Representative western blot bands **(A)** and quantification of the protein levels of NLRP3 **(B)**, caspase-1 **(C)**, cleaved caspase-1 **(D)**, GSDMD **(E)**, and cleaved GSDMD **(F)** (n = 6 per group). Representative immunofluorescence microphotographs of NLRP3 staining **(G)** in the ipsilateral hemisphere. Scale bar = 50 μm. Measurement of NLRP3 staining in the ipsilateral hemisphere **(H)** (n = 6 per group). Values are presented as the mean ± SD. ^@^ *P* < 0.05 compared with sham group, *P < 0.05 compared with SAH + vehicle group, ^&^ *P* < 0.05 compared with SAH + 20mg/kg LDC7559 group.

NLRP3 inflammasome could trigger caspase-1 activation and subsequently induce IL-1β and IL-18 maturation. Additionally, activated caspase-1 exerts an important role in the intersection of the pyroptosis and the apoptosis pathways. Pyroptosis is characterized by GSDMD-mediated. Caspase-1 has been demonstrated not only to modulate GSDMD activation but also affect caspase-3 activation-mediated neuronal apoptosis. We further evaluated the effects of LDC7559 on NLRP3/caspase1/GSDMD signaling after SAH. It revealed that SAH significantly induced the activation of NLRP3/caspase1/GSDMD signaling. In contrast, LDC7559 treatment markedly inhibited the expressions of GSDMD and cleaved GSDMD after SAH (**Figures 4A–H**). However, LDC7559 did not significantly affect the levels of NLRP3 and caspase1. Nigericin was further used to activate NLRP3 inflammasome signaling. As expected, nigericin significantly induced NLRP3/caspase1/GSDMD signaling and abated the inhibitory effects of LDC7559 on GSDMD after SAH (**Figures 4A–H**).

### 3.5 LDC7559 treatment improved cell viability and ameliorated neuroinflammation *in vitro*

**Figure 5:**
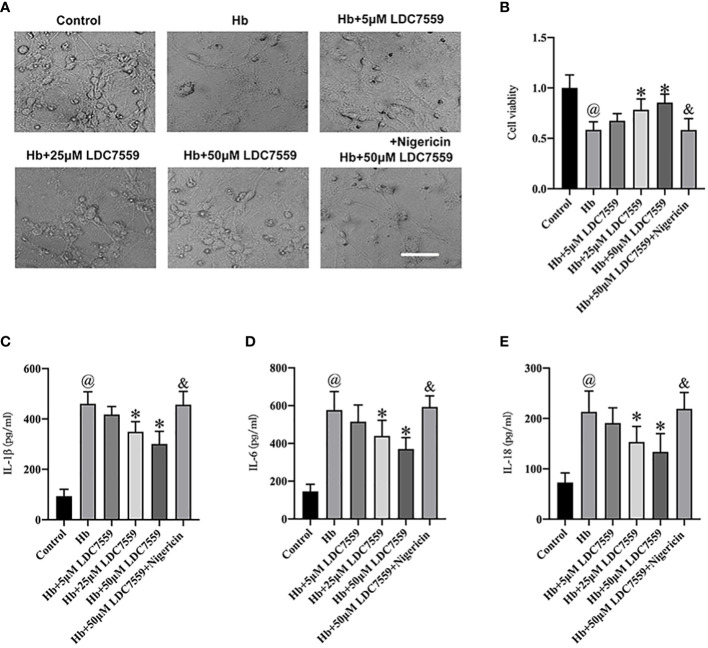
LDC7559 improved cell viability and reduced inflammatory response *in vitro* experiments. Phase-contrast photomicrographs of cultured neurons in all groups **(A)**. Scale bar = 50 μm. Changes of cell viability were determined by CCK-8 assay **(B)** (n = 6 per group). ELISA kits were used to determine the levels of IL-1β **(C)**, IL-6 **(D)**, and IL-18 **(E)** (n = 6 per group). Values are presented as the mean ± SD. ^@^ *P* < 0.05 compared with control group, *P < 0.05 compared with Hb group, ^&^ *P* < 0.05 compared with Hb + 50μM LDC7559 group.

We further verified the beneficial effects of LDC7559 on neuroinflammation and cell viability *in vitro*. A transwell co-culture system was established in this experiment. It showed that the neuronal cell viability was evidently decreased after SAH insults (**Figures 5A, B**). In addition, Hb stimulation significantly induced proinflammatory cytokines release, including IL-1β, IL-6, and IL-18 (**Figures 5C–E**). In contrast, LDC7559 dose-dependently inhibited the levels of proinflammatory cytokines and further improved cell viability. However, the beneficial effects of LDC7559 *in vitro* were abated by nigericin (**Figures 5A–E**).

### 3.6 LDC7559 mitigated neuronal pyroptosis and neuronal apoptosis *in vitro*

**Figure 6:**
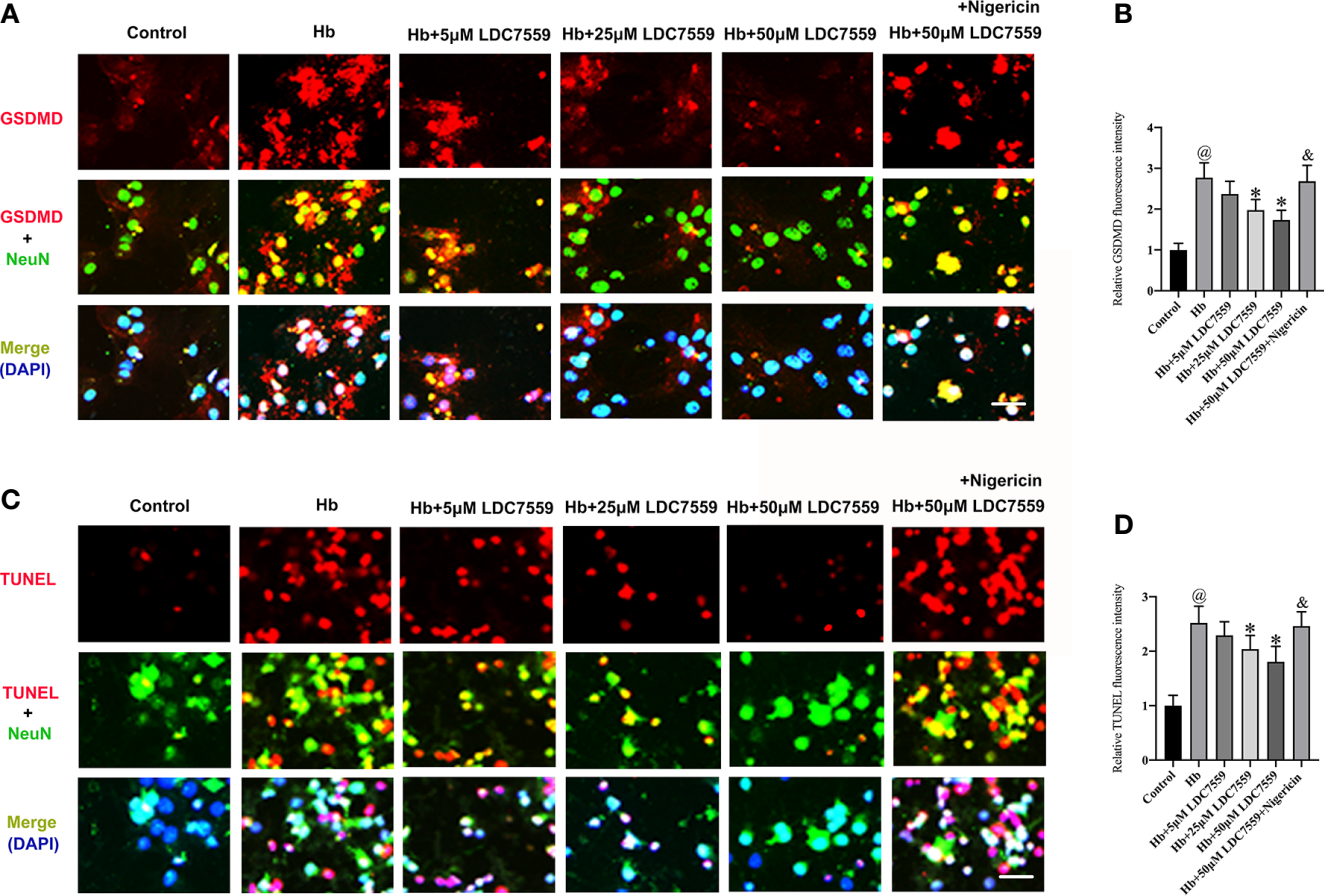
LDC7559 reduced neuronal apoptosis and pyroptosis *in vitro*. Representative immunofluorescence microphotographs of GSDMD-positive neurons **(A)**. Scale bar = 50 μm. Measurement of GSDMD-positive neurons in all groups **(B)**. Representative immunofluorescence microphotographs of TUNEL-positive neurons **(C)**. Scale bar = 50 μm. Measurement of TUNEL-positive neurons in all groups **(D)**. Values are presented as the mean ± SD. ^@^ *P* < 0.05 compared with control group, *P < 0.05 compared with Hb group, ^&^ *P* < 0.05 compared with Hb + 50μM LDC7559 group.

Whether LDC7559 could inhibit neuronal apoptosis and pyroptosis *in vitro* remains unknown. We further evaluated the influence of LDC7559 on neuronal apoptosis and pyroptosis *in vitro*. It showed that the numbers of GSDMD-positive and TUNEL-positive neurons were significantly induced after Hb stimulation, whereas LDC7559 rescued the high levels of neuronal apoptosis and pyroptosis (**Figures 6A–D**). In contrast, NLRP3 inflammasome activator nigericin further aggravated neuronal apoptosis and pyroptosis and abated the beneficial effects of LDC7559 (**Figures 6A–D**). Overall, these findings indicated that LDC7559 exerted a protective effect on SAH-induced neuroinflammation and neuronal apoptosis and pyroptosis.

## 4 Discussion

According to previous studies, LDC7559 has been verified as a selective pyroptosis inhibitor (21). In models of TBI, LDC7559 significantly inhibited GSDMD and cleaved GSDMD in microglia. Meanwhile, LDC7559 improved neurobehavioral function and mitigated brain tissue loss after TBI (21). Consistent with previous reports, our data indicated that LDC7559 dramatically inhibited microglial activation and the pro-inflammatory cytokines release after SAH. In addition, LDC7559 suppressed neuronal pyroptosis and apoptosis after SAH. Concomitant with the decreased inflammatory insults and neuronal death, LDC7559 significantly improved neurological outcomes after SAH. Mechanistically, LDC7559 inhibited GSDMD activation and GSDMD-N generation after SAH. In contrast, NLRP3 inflammasome activation by nigericin further aggravated inflammatory insults, neuronal apoptosis and pyroptosis. Meanwhile, the beneficial effects of LDC7559 against SAH were abated by nigericin.

Mounting evidence has indicated that neuroinflammation plays a critical role in SAH-triggered brain damage. After SAH, ischemia with its associated cell death cause inflammatory cells infiltration, microglial activation and proinflammatory cytokines release in the brain (2). Inflammasomes participate in the inflammatory responses after SAH. Among them, NLRP3 inflammasome has been extensively studied in different neurological diseases (28–30). NLRP3 inflammasome could trigger caspase-1 activation and subsequently induce the maturation of IL-1β and IL-18. In addition, activated caspase-1 could lead to pyroptotic cell death and apoptotic cell death (15). In central nervous system (CNS) diseases, numerous studies have demonstrated that inhibiting NLRP3 could ameliorate neural cell death and neuroinflammation (31, 32).

Pyroptosis is a recently discovered cell death form, which can be driven by inflammatory processes. Recently, some scholars have proved that pyroptosis is characterized by GSDMD-mediated, rather than caspase-1-mediated (33, 34). Shi et al. reported that pyroptosis cannot be initiated by canonical inflammasome ligands in GSDMD-deficient cells (33). Caspase-1 activation could cleave GSDMD and generate GSDMD-N. GSDMD-N could rupture cell membranes and trigger pyroptosis (35). In CNS diseases models including TBI and SAH, GSDMD and GSDMD-N were enhanced after injury (35, 36). For example, yuan et al. indicated that inhibition of absent in melanoma 2 (AIM2) inflammasome alleviated GSDMD-mediated pyroptosis after SAH. In addition, caspase-1 deficiency could mitigate both apoptosis and GSDMD-mediated pyroptosis after SAH (35). In this study, we also observed that NLRP3 inflammasome, cleaved caspase-1, GSDMD and GSDMD-N were upregulated after SAH. Concomitant with the activation of NLRP3/caspase-1/GSDMD signaling, microglial activation and the pro-inflammatory cytokines release were markedly induced after SAH. In addition, both neuronal apoptosis and pyroptosis were significantly triggered after SAH. These indicated that targeting NLRP3/caspase-1/GSDMD signaling might be a feasible method for treating SAH.

LDC7559 is a newly discovered small molecule. It can selectively inhibit GSDMD and bind to the GSDMD-N domain, thereby suppressing pyroptosis (21). A previous study indicated that LDC7559 could provide beneficial effects against GSDMD-dependent pyroptosis and microglial activation in TBI mice model (21). However, little is known about the effects of LDC7559 on SAH-induced brain damage. Recently, mounting evidence indicates that pyroptosis exerts a critical role in various brain injuries (37–39). Moreover, targeting pyroptosis has achieved positive results in preclinical research. We suspected that LDC7559 might be a promising approach for SAH. As expected, our data revealed that LDC7559 inhibited microglial activation and GSDMD-dependent pyroptosis after SAH. In addition, we found that LDC7559 also reduced neuronal apoptosis after SAH. In fact, GSDMD is the downstream target of caspase-1. Our data showed that LDC7559 did not affect caspase-1 expression. We speculated that the reduced neuronal apoptosis might be related with the decreased neuroinflammation. However, it should be noted that the caspase-1 activation is closely associated with inflammatory response. NLRP3 inflammasome activation by nigericin further increased caspase-1 activation and abated the beneficial effects of LDC7559 on SAH. These suggest that the processes of pyroptosis are complicated. The specific mechanism of pyroptosis after SAH needs further exploration.

Our present study has some limitations. Firstly, we cannot exclude other molecular targets involved in the beneficial effects of LDC7559 against SAH. Besides modulation of GSDMD-mediated pyroptosis, LDC7559 can suppress nicotinamide adenine dinucleotide phosphate oxidase 2 (NOX2)-dependent oxidative stress (40). NOX2-mediated oxidative stress also plays an important role in the pathophysiology of SAH (41). Secondly, the long-term beneficial effects of LDC7559 after SAH remain unknown. In addition, the gender-specific optimal dose, therapeutic time window, and the administration route and frequency of LDC7559 should be further determined before clinical application.

## 5 Conclusion

Taken together, our study demonstrated, for the first time, that LDC7559 mitigated microglial activation and neuronal pyroptosis by inhibiting GSDMD after SAH both *in vivo* and *in vitro*. LDC7559 might be a feasible candidate for SAH.

## Data availability statement

The original contributions presented in the study are included in the article/**Supplementary Material**. Further inquiries can be directed to the corresponding authors.

## Ethics statement

The animal study was reviewed and approved by the Second Affiliated Hospital of Fujian Medical University.

## Author contributions

Conceptualization, WC, ZW, CX, XC. Methodology, ZW, JL, JY, YZ, WL, ZF, and JC performed experiments. Data analysis, WC, ZW, JL, and CX. Writing—review and editing, WC, ZW, CX, and XC. All authors contributed to the article and approved the submitted version.

## References

[1] Macdonald RL. Age and outcome after aneurysmal subarachnoid haemorrhage. J Neurol Neurosurg Psychiatry (2021) 92:1143. doi: 10.1136/jnnp-2021-326920 34187868

[2] Heinz R Brandenburg S Nieminen-Kelha M Kremenetskaia I Boehm-Sturm P Vajkoczy P. Microglia as target for anti-inflammatory approaches to prevent secondary brain injury after subarachnoid hemorrhage (sah). J Neuroinflamm (2021) 18:36. doi: 10.1186/s12974-021-02085-3 PMC7847606 33516246

[3] Geraghty JR Davis JL Testai FD. Neuroinflammation and microvascular dysfunction after experimental subarachnoid hemorrhage: Emerging components of early brain injury related to outcome. Neurocrit Care (2019) 31:373–89. doi: 10.1007/s12028-019-00710-x PMC6759381 31012056

[4] Al-Tamimi YZ Bhargava D Orsi NM Teraifi A Cummings M Ekbote UV. Compartmentalisation of the inflammatory response following aneurysmal subarachnoid haemorrhage. Cytokine (2019) 123:154778. doi: 10.1016/j.cyto.2019.154778 31323526

[5] Lin W Yao H Lai J Zeng Y Guo X Lin S. Cycloastragenol confers cerebral protection after subarachnoid hemorrhage by suppressing oxidative insults and neuroinflammation *via* the sirt1 signaling pathway. Oxid Med Cell Longev (2022) 2022:3099409. doi: 10.1155/2022/3099409 35693703 PMC9184193

[6] Zeng Y Fang Z Lai J Wu Z Lin W Yao H. Activation of sirtuin-1 by pinocembrin treatment contributes to reduced early brain injury after subarachnoid hemorrhage. Oxid Med Cell Longev (2022) 2022:2242833. doi: 10.1155/2022/2242833 36439686 PMC9683949

[7] Chen J Wong GKC. Microglia accumulation and activation after subarachnoid hemorrhage. Neural Regener Res (2021) 16:1531–2. doi: 10.4103/1673-5374.303028 PMC8323694 33433468

[8] Coulibaly AP Provencio JJ. Aneurysmal subarachnoid hemorrhage: An overview of inflammation-induced cellular changes. Neurotherapeutics (2020) 17:436–45. doi: 10.1007/s13311-019-00829-x PMC7283430 31907877

[9] Song D Yeh CT Wang J Guo F. Perspectives on the mechanism of pyroptosis after intracerebral hemorrhage. Front Immunol (2022) 13:989503. doi: 10.3389/fimmu.2022.989503 36131917 PMC9484305

[10] Jorgensen I Miao EA. Pyroptotic cell death defends against intracellular pathogens. Immunol Rev (2015) 265:130–42. doi: 10.1111/imr.12287 PMC4400865 25879289

[11] Humphries F Shmuel-Galia L Ketelut-Carneiro N Li S Wang B Nemmara VV. Succination inactivates gasdermin d and blocks pyroptosis. Science (2020) 369:1633–7. doi: 10.1126/science.abb9818 PMC8744141 32820063

[12] Coll RC Schroder K Pelegrin P. Nlrp3 and pyroptosis blockers for treating inflammatory diseases. Trends Pharmacol Sci (2022) 43:653–68. doi: 10.1016/j.tips.2022.04.003 35513901

[13] Wang C Yang T Xiao J Xu C Alippe Y Sun K. Nlrp3 inflammasome activation triggers gasdermin d-independent inflammation. Sci Immunol (2021) 6:eabj3859. doi: 10.1126/sciimmunol.abj3859 34678046 PMC8780201

[14] Li S Sun Y Song M Song Y Fang Y Zhang Q. Nlrp3/caspase-1/gsdmd-mediated pyroptosis exerts a crucial role in astrocyte pathological injury in mouse model of depression. JCI Insight (2021) 6:e146852. doi: 10.1172/jci.insight.146852 34877938 PMC8675200

[15] Zhang XS Lu Y Li W Tao T Wang WH Gao S. Cerebroprotection by dioscin after experimental subarachnoid haemorrhage *via* inhibiting nlrp3 inflammasome through sirt1-dependent pathway. Br J Pharmacol (2021) 178:3648–66. doi: 10.1111/bph.15507 33904167

[16] Zhang ZH Liu JQ Hu CD Zhao XT Qin FY Zhuang Z. Luteolin confers cerebroprotection after subarachnoid hemorrhage by suppression of nlpr3 inflammasome activation through nrf2-dependent pathway. Oxid Med Cell Longev (2021) 2021:5838101. doi: 10.1155/2021/5838101 34777689 PMC8589510

[17] Dodd WS Noda I Martinez M Hosaka K Hoh BL. Nlrp3 inhibition attenuates early brain injury and delayed cerebral vasospasm after subarachnoid hemorrhage. J Neuroinflamm (2021) 18:163. doi: 10.1186/s12974-021-02207-x PMC8293512 34284798

[18] Yamaguchi T Miyamoto T Shikata E Yamaguchi I Shimada K Yagi K. Activation of the nlrp3/il-1beta/mmp-9 pathway and intracranial aneurysm rupture associated with the depletion of eralpha and sirt1 in oophorectomized rats. J Neurosurg (2022) 20:191–8. doi: 10.3171/2022.4.JNS212945 35594890

[19] Sollberger G Choidas A Burn GL Habenberger P Di Lucrezia R Kordes S. Gasdermin d plays a vital role in the generation of neutrophil extracellular traps. Sci Immunol (2018) 3:eaar6689. doi: 10.1126/sciimmunol.aar6689 30143555

[20] Pandeya A Li L Li Z Wei Y. Gasdermin d (gsdmd) as a new target for the treatment of infection. Medchemcomm (2019) 10:660–7. doi: 10.1039/C9MD00059C PMC6533889 31191857

[21] Yu E Zhang E Lv X Yan L Lin Z Siaw-Debrah F. Ldc7559 exerts neuroprotective effects by inhibiting gsdmd-dependent pyroptosis of microglia in mice with traumatic brain injury. J Neurotrauma (2022). doi: 10.1089/neu.2021.0318 35920115

[22] Huang Y Guo Y Huang L Fang Y Li D Liu R. Kisspeptin-54 attenuates oxidative stress and neuronal apoptosis in early brain injury after subarachnoid hemorrhage in rats *via* gpr54/arrb2/akt/gsk3beta signaling pathway. Free Radic Biol Med (2021) 171:99–111. doi: 10.1016/j.freeradbiomed.2021.05.012 33989759 PMC8388553

[23] Zhang X Lu Y Wu Q Dai H Li W Lv S. Astaxanthin mitigates subarachnoid hemorrhage injury primarily by increasing sirtuin 1 and inhibiting the toll-like receptor 4 signaling pathway. FASEB J (2019) 33:722–37. doi: 10.1096/fj.201800642RR 30048156

[24] Shao A Gao S Wu H Xu W Pan Y Fang Y. Melatonin ameliorates hemorrhagic transformation *via* suppression of ros-induced nlrp3 activation after cerebral ischemia in hyperglycemic rats. Oxid Med Cell Longev (2021) 2021:6659282. doi: 10.1155/2021/6659282 33777317 PMC7972845

[25] Liu GJ Tao T Zhang XS Lu Y Wu LY Gao YY. Resolvin d1 attenuates innate immune reactions in experimental subarachnoid hemorrhage rat model. Mol Neurobiol (2021) 58:1963–77. doi: 10.1007/s12035-020-02237-1 33411245

[26] Alexander SPH Roberts RE Broughton BRS Sobey CG George CH Stanford SC. Goals and practicalities of immunoblotting and immunohistochemistry: A guide for submission to the british journal of pharmacology. Br J Pharmacol (2018) 175:407–11. doi: 10.1111/bph.14112 PMC5773976 29350411

[27] Sugawara T Ayer R Jadhav V Zhang JH. A new grading system evaluating bleeding scale in filament perforation subarachnoid hemorrhage rat model. J Neurosci Methods (2008) 167:327–34. doi: 10.1016/j.jneumeth.2007.08.004 PMC2259391 17870179

[28] Milner MT Maddugoda M Gotz J Burgener SS Schroder K. The nlrp3 inflammasome triggers sterile neuroinflammation and alzheimer's disease. Curr Opin Immunol (2021) 68:116–24. doi: 10.1016/j.coi.2020.10.011 33181351

[29] O'Brien WT Pham L Symons GF Monif M Shultz SR McDonald SJ. The nlrp3 inflammasome in traumatic brain injury: Potential as a biomarker and therapeutic target. J Neuroinflamm (2020) 17:104. doi: 10.1186/s12974-020-01778-5 PMC7137518 32252777

[30] Heneka MT McManus RM Latz E. Inflammasome signalling in brain function and neurodegenerative disease. Nat Rev Neurosci (2018) 19:610–21. doi: 10.1038/s41583-018-0055-7 30206330

[31] Ren H Kong Y Liu Z Zang D Yang X Wood K. Selective nlrp3 (pyrin domain-containing protein 3) inflammasome inhibitor reduces brain injury after intracerebral hemorrhage. Stroke (2018) 49:184–92. doi: 10.1161/STROKEAHA.117.018904 PMC5753818 29212744

[32] Marín-Aguilar F Lechuga-Vieco AV Alcocer-Gómez E Castejón-Vega B Lucas J Garrido C. Nlrp3 inflammasome suppression improves longevity and prevents cardiac aging in male mice. Aging Cell (2019) 19:e13050. doi: 10.1111/acel.13050 31625260 PMC6974709

[33] Shi J Gao W Shao F. Pyroptosis: Gasdermin-mediated programmed necrotic cell death. Trends Biochem Sci (2017) 42:245–54. doi: 10.1016/j.tibs.2016.10.004 27932073

[34] Shi J Zhao Y Wang K Shi X Wang Y Huang H. Cleavage of gsdmd by inflammatory caspases determines pyroptotic cell death. Nature (2015) 526:660–5. doi: 10.1038/nature15514 26375003

[35] Yuan B Zhou XM You ZQ Xu WD Fan JM Chen SJ. Inhibition of aim2 inflammasome activation alleviates gsdmd-induced pyroptosis in early brain injury after subarachnoid haemorrhage. Cell Death Dis (2020) 11:76. doi: 10.1038/s41419-020-2248-z 32001670 PMC6992766

[36] Liu W Chen Y Meng J Wu M Bi F Chang C. Ablation of caspase-1 protects against tbi-induced pyroptosis *in vitro* and *in vivo* . J Neuroinflamm (2018) 15:48. doi: 10.1186/s12974-018-1083-y PMC5817788 29458437

[37] Corcoran SE Halai R Cooper MA. Pharmacological inhibition of the nod-like receptor family pyrin domain containing 3 inflammasome with mcc950. Pharmacol Rev (2021) 73:968–1000. doi: 10.1124/pharmrev.120.000171 34117094

[38] Chen S Zuo Y Huang L Sherchan P Zhang J Yu Z. The mc4 receptor agonist ro27-3225 inhibits nlrp1-dependent neuronal pyroptosis *via* the ask1/jnk/p38 mapk pathway in a mouse model of intracerebral haemorrhage. Br J Pharmacol (2019) 176:1341–56. doi: 10.1111/bph.14639 PMC6468256 30811584

[39] Bobinger T Burkardt P B Huttner H Manaenko A. Programmed cell death after intracerebral hemorrhage. Curr Neuropharmacol (2018) 16:1267–81. doi: 10.2174/1570159X15666170602112851 PMC6251052 28571544

[40] Amara N Cooper MP Voronkova MA Webb BA Lynch EM Kollman JM. Selective activation of pfkl suppresses the phagocytic oxidative burst. Cell (2021) 184:4480–4494 e4415. doi: 10.1016/j.cell.2021.07.004 34320407 PMC8802628

[41] Zhang L Li Z Feng D Shen H Tian X Li H. Involvement of nox2 and nox4 nadph oxidases in early brain injury after subarachnoid hemorrhage. Free Radic Res (2017) 51:316–28. doi: 10.1080/10715762.2017.1311015 28330417

