# Supplementary material for: LDC7559 inhibits microglial activation and GSDMD-dependent pyroptosis after subarachnoid hemorrhage
Source: Front Immunol. 2023 Mar 29;14:1117310. doi: 10.3389/fimmu.2023.1117310 (PMC10090682; doi:10.3389/fimmu.2023.1117310)
Supplement: Supplementary file 1 [file Table_1.docx]

The antibodies used in the study.

| **Antibody** | **Item number** | **Company** |
| --- | --- | --- |
| Anti-Iba1 | SC-98468 | Santa Cruz |
| Anti-GSDMD | orb593258 | Biorbyt |
| Anti-NLRP3 | SC-66846 | Santa Cruz |
| Anti-NeuN | MAB-377 | MilliporeSigma |
| Anti-Caspase1 | Ab108362 | Abcam |
| Anti-Cleaved caspase1 | 22915-1-AP | Proteintech |
| Anti-GSDMD-N | NBP2-33422 | Novus |
| Anti-β-actin | AP0060 | Bioworld |
